# Beneficial Effects of Canagliflozin in Combination with Pioglitazone on Insulin Sensitivity in Rodent Models of Obese Type 2 Diabetes

**DOI:** 10.1371/journal.pone.0116851

**Published:** 2015-01-23

**Authors:** Yoshinori Watanabe, Keiko Nakayama, Nobuhiko Taniuchi, Yasushi Horai, Chiaki Kuriyama, Kiichiro Ueta, Kenji Arakawa, Takaaki Senbonmatsu, Masaharu Shiotani

**Affiliations:** 1 Pharmacology Research Laboratories II, Research Division, Mitsubishi Tanabe Pharma Corporation, Saitama, Japan; 2 Safety Research Laboratory, Research Division, Mitsubishi Tanabe Pharma Corporation, Saitama, Japan; 3 Department of Pharmacology, Saitama Medical University, Saitama, Japan; Max-Delbrück Center for Molecular Medicine (MDC), GERMANY

## Abstract

**Background:**

Despite its insulin sensitizing effects, pioglitazone may induce weight gain leading to an increased risk of development of insulin resistance. A novel sodium glucose co-transporter 2 (SGLT2) inhibitor, canagliflozin, provides not only glycemic control but also body weight reduction through an insulin-independent mechanism. The aim of this study was to investigate the combined effects of these agents on body weight control and insulin sensitivity.

**Methods:**

Effects of combination therapy with canagliflozin and pioglitazone were evaluated in established diabetic KK-Ay mice and prediabetic Zucker diabetic fatty (ZDF) rats.

**Results:**

In the KK-Ay mice, the combination therapy further improved glycemic control compared with canagliflozin or pioglitazone monotherapy. Furthermore, the combination significantly attenuated body weight and fat gain induced by pioglitazone and improved hyperinsulinemia. In the ZDF rats, early intervention with pioglitazone monotherapy almost completely prevented the progressive development of hyperglycemia, and no further improvement was observed by add-on treatment with canagliflozin. However, the combination significantly reduced pioglitazone-induced weight gain and adiposity and improved the Matsuda index, suggesting improved whole-body insulin sensitivity.

**Conclusions:**

Our study indicates that combination therapy with canagliflozin and pioglitazone improves insulin sensitivity partly by preventing glucotoxicity and, at least partly, by attenuating pioglitazone-induced body weight gain in two different obese diabetic animal models. This combination therapy may prove to be a valuable option for the treatment and prevention of obese type 2 diabetes.

## Introduction

Obesity is a global pandemic and one of the most important public health concerns in many countries [1,2]. Medical costs for people with obesity are approximately 30% greater than for people with normal weights, because obesity is related to serious health problems such as diabetes, hypertension, and other components of metabolic syndrome [3]. In particular, it has been well recognized that obesity is a risk factor for developing insulin resistance and type 2 diabetes [4–6]. Considering the increasing prevalence of obesity, it is predicted that the total number of people with diabetes in the world will rise to more than 366 million by 2030 [7].

At present, many types of pharmacological agents are available for the treatment of type 2 diabetes [8]. However, almost half of the diabetic adults in the United States still do not meet the targets for glycemic control [9]. It is important to carefully assess the risk–benefit profile before selecting the appropriate pharmacological therapy [10]. Weight gain is one of the side effects of the thiazolidinedione (TZD) insulin sensitizer, pioglitazone [11]. On the other hand, canagliflozin, the first-in-class sodium glucose co-transporter 2 (SGLT2) inhibitor available in the United States, not only lowers blood glucose through an insulin-independent mechanism but also reduces body weight [12,13] via excretion of excess blood glucose into the urine [14–16]. Position statements from the American Diabetes Association and the European Association for the Study of Diabetes recommend combination therapy with two or three drugs if monotherapy does not achieve target HbA1c levels [17]. It was recently reported that add-on treatment with canagliflozin significantly improved glycemic control and reduced body weight in patients with type 2 diabetes inadequately controlled by metformin and pioglitazone [18]. However, little is known about the effects of combination therapy with an SGLT2 inhibitor and TZD on insulin sensitivity.

In this study, we investigated how the combination of canagliflozin and pioglitazone affected body weight control and insulin sensitivity both in established diabetic KK-A^y^ mice and prediabetic Zucker diabetic fatty (ZDF) rats.

## Materials and Methods

### Animals

All experimental procedures were approved by the institutional animal care and use committee of Mitsubishi Tanabe Pharma Corporation (Osaka, Japan) and animal care was conducted in accordance with institutional guidelines. Male KK-A^y^ mice were purchased from CLEA Japan (Tokyo, Japan), and male Zucker diabetic fatty (ZDF) rats and Zucker lean (ZL) rats were purchased from Charles River Laboratories Japan (Tokyo, Japan). The animals were individually housed in plastic cages with ad libitum access to standard chow (CRF-1; Oriental Yeast Co., Ltd., Tokyo, Japan) and tap water. The animal rooms were controlled for temperature (23 ± 3°C), humidity (55 ± 20%), and a 12-h light/dark cycle. These animals were acclimated for 1 week prior to the start of the studies.

### Drugs

Canagliflozin and pioglitazone hydrochloride were synthesized at the Medicinal Chemistry Laboratory of Mitsubishi Tanabe Pharma Corporation.

### Diabetes intervention study in KK-A^y^ mice

Thirteen-week-old established diabetic male KK-A^y^ mice were treated with canagliflozin (0.01% w/w food admixture), pioglitazone (0.01% w/w food admixture), and their combination (canagliflozin 0.01% w/w and pioglitazone 0.01% w/w food admixture) for 2 weeks. Average doses were estimated from body weight and daily food intake. Body weight was measured at least once a week. Accumulated food intake was calculated by summing the daily food intake. Plasma glucose and insulin were measured weekly. Blood samples were collected from the tail vein using a heparinized micro-capillary tube, and plasma was obtained by centrifugation at 1800 *g* for 15 min at 4°C. Twenty-four-hour urine samples were collected on days 14–15 using metabolic cages for the measurement of urinary glucose excretion (UGE). At the end of the study, body composition and histology of mesenteric adipose tissue were analyzed as described below.

### Body composition of KK-A^y^ mice

Fat mass and lean mass (whole body) were evaluated at the end of the study using dual-energy X-ray absorptiometry (DEXA) (PIXImus2; GE Medical Systems, Inc., Madison, WI, USA). All images were analyzed using PIXImus2 software. In addition, computed tomography (CT) was performed using an experimental animal CT system (LaTheta LCT-100; Hitachi-Aloka Medical, Ltd., Tokyo, Japan). CT images (3-mm thick) were acquired from the entire abdominal region of each animal under pentobarbital anesthesia. Quantitative assessment of the subcutaneous and visceral fat was performed using LaTheta software. Subcutaneous and visceral fat tissues were distinguished by manual tracing of the abdominal wall in each of the sections.

### Histological analysis of adipose tissue in KK-A^y^ mice

Mesenteric adipose tissues of KK-A^y^ mice were isolated and immediately fixed in 10% neutralized buffered formalin. Formalin-fixed samples were embedded in paraffin, cut into 4-μm sections, and stained with elastic van Gieson without nuclear staining. Whole slide digital images of the adipose tissue were obtained using Scan-Scope XT (Aperio Technologies, Inc., Vista, CA, USA). Five images were extracted in the area of 1,000,000 μm^2^ from the whole slide images using Image-Scope (Aperio Technologies, Inc.). Each image was converted via 8-bit gray scale processing and binarization. The adipocyte area was then measured (excluding any area of <100 μm^2^) using Image-Pro Plus (Media Cybernetics, Inc., Bethesda, MD, USA). After manually excluding all non-adipocyte objects, such as intercellular spaces and blood vessels, a standard measure of 100 adipocytes per image was established.

### Diabetes prevention study in prediabetic ZDF rats

Seven-week-old prediabetic male ZDF rats were treated by oral gavage with canagliflozin (daily dose of 10 mg/kg), pioglitazone (daily dose of 10 mg/kg), and their combination at the same dosages for 6 weeks. All drugs were suspended in 0.5% w/v hydroxypropyl methylcellulose (HPMC) at a concentration of 2 mg/mL. The drugs or vehicle (0.5% HPMC) were administered orally at a volume of 5 mL/kg body weight using a disposable feeding needle (Fuchigami Kikai, Kyoto, Japan) and syringe (Terumo, Tokyo, Japan). Accumulated food intake was calculated by summing the weekly food intake. Body weight, plasma glucose, plasma insulin, and HbA1c were measured every 2 weeks. Blood samples were collected from the tail vein, and plasma was obtained as described above. After the 6-week treatment period, overnight fasted animals were subjected to an oral glucose tolerance test (OGTT) 2 days after the last dosing. Blood samples were collected at 0, 15, 30, 60, and 120 min after oral administration of glucose solution (2 g/kg/5 mL). Whole-body insulin sensitivity during OGTT was assessed according to the Matsuda index, with values derived from fasting plasma glucose (FPG), fasting plasma insulin (FPI), mean plasma glucose during OGTT (MPG), and mean plasma insulin during OGTT (MPI), as previously reported [19]: Matsuda index = 10,000 / √{(FPG × FPI) × (MPG × MPI)}

After OGTT, the rats were refed and retreated for 3 days. At the end of the study, the rats were euthanized by whole blood collection from the abdominal aorta under isoflurane anesthesia. Mesenteric, epididymal, and perirenal fat, as well as the pancreas, were quickly removed and weighed. The pancreas was immediately frozen in liquid nitrogen and stored at −80°C to measure insulin.

### Analytical procedures

Plasma and urinary glucose levels were measured using the glucose CII-test Wako (Wako Pure Chemical Industries, Ltd., Osaka, Japan). Pancreatic insulin was extracted by an acid–ethanol solution, as previously reported [14]. Plasma and pancreatic insulin levels were measured by ELISA, using the Ultra Sensitive Mouse Insulin ELISA Kit or Ultra Sensitive Rat Insulin ELISA Kit (Morinaga Institute of Biological Science, Inc., Yokohama, Japan). HbA1c in whole blood was measured using the DCA2000 analyzer (Bayer Medical, Ltd., Tokyo, Japan).

### Statistical analysis

Data are expressed as mean ± standard error of the mean (SEM). Significant differences between groups were analyzed using one-way analysis of variance (ANOVA), followed by the Student’s *t*-test, as previously reported [20,21]. Probabilities less than 5% (*P* < 0.05) were considered significant. All statistical analyses were performed using the SAS system (SAS Institute Japan, Tokyo, Japan).

## Results

### Glycemic control in KK-A^y^ mice

**Figure 1:**
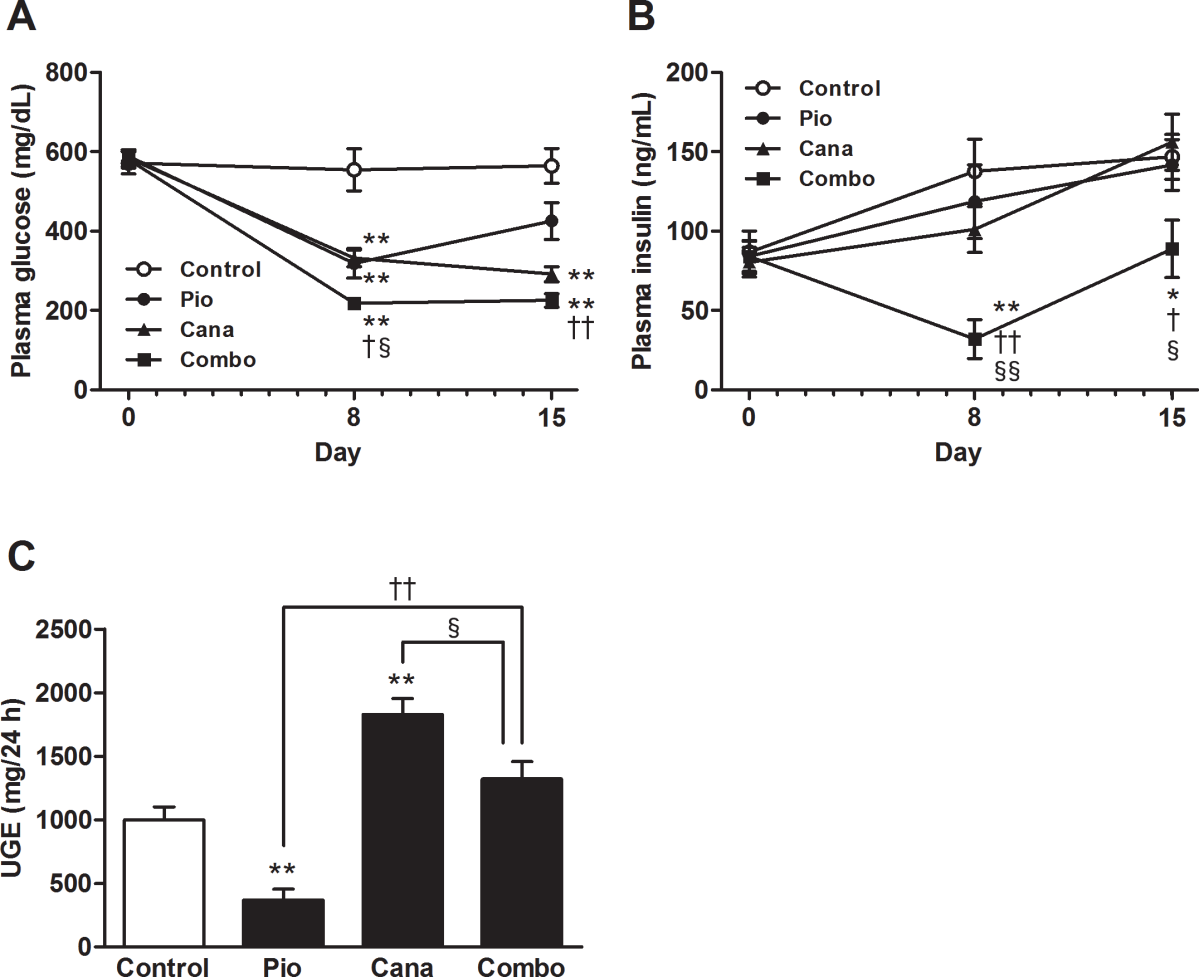
Glycemic control and urinary glucose excretion (UGE) in KK-A^y^ mice. A: Plasma glucose, B: Plasma insulin, and C: UGE. Canagliflozin (Cana; 0.01% w/w food admixture), pioglitazone (Pio; 0.01% w/w food admixture), and their combination (Combo) were administered for 2 weeks. UGE was measured on days 14–15. Data are expressed as mean ± SEM (n = 7–8). * *P* < 0.05, ** *P* < 0.01 vs. control, † *P* < 0.05, †† *P* < 0.01 vs. Pio, § *P* < 0.05, §§ *P* < 0.01 vs. Cana.

The effects of the combination of canagliflozin and pioglitazone on glycemic control were evaluated for 2 weeks in the established diabetic KK-A^y^ mice. The average daily dose of the drugs (calculated from food intake and body weight) was as follows; pioglitazone 0.01% w/w food admixture, 13.0 mg/kg; canagliflozin 0.01% w/w food admixture, 16.6 mg/kg; and the combination of canagliflozin 0.01% w/w and pioglitazone 0.01% w/w food admixture, canagliflozin 15.1 mg/kg and pioglitazone 15.1 mg/kg. Canagliflozin or pioglitazone monotherapy significantly improved glycemic control, which improved further with the drugs in combination (Fig. 1A). Neither type of monotherapy affected plasma insulin levels, but the combination significantly ameliorated hyperinsulinemia (Fig. 1B). Although remarkable UGE was observed in the control KK-A^y^ mice, canagliflozin monotherapy significantly enhanced UGE (Fig. 1C). UGE with the combination therapy was also significantly higher than that with pioglitazone monotherapy. However, UGE with the combination therapy was significantly lower than that with canagliflozin monotherapy, probably reflecting the contribution of pioglitazone to glycemic control.

### Body weight and adiposity in KK-A^y^ mice

**Figure 2:**
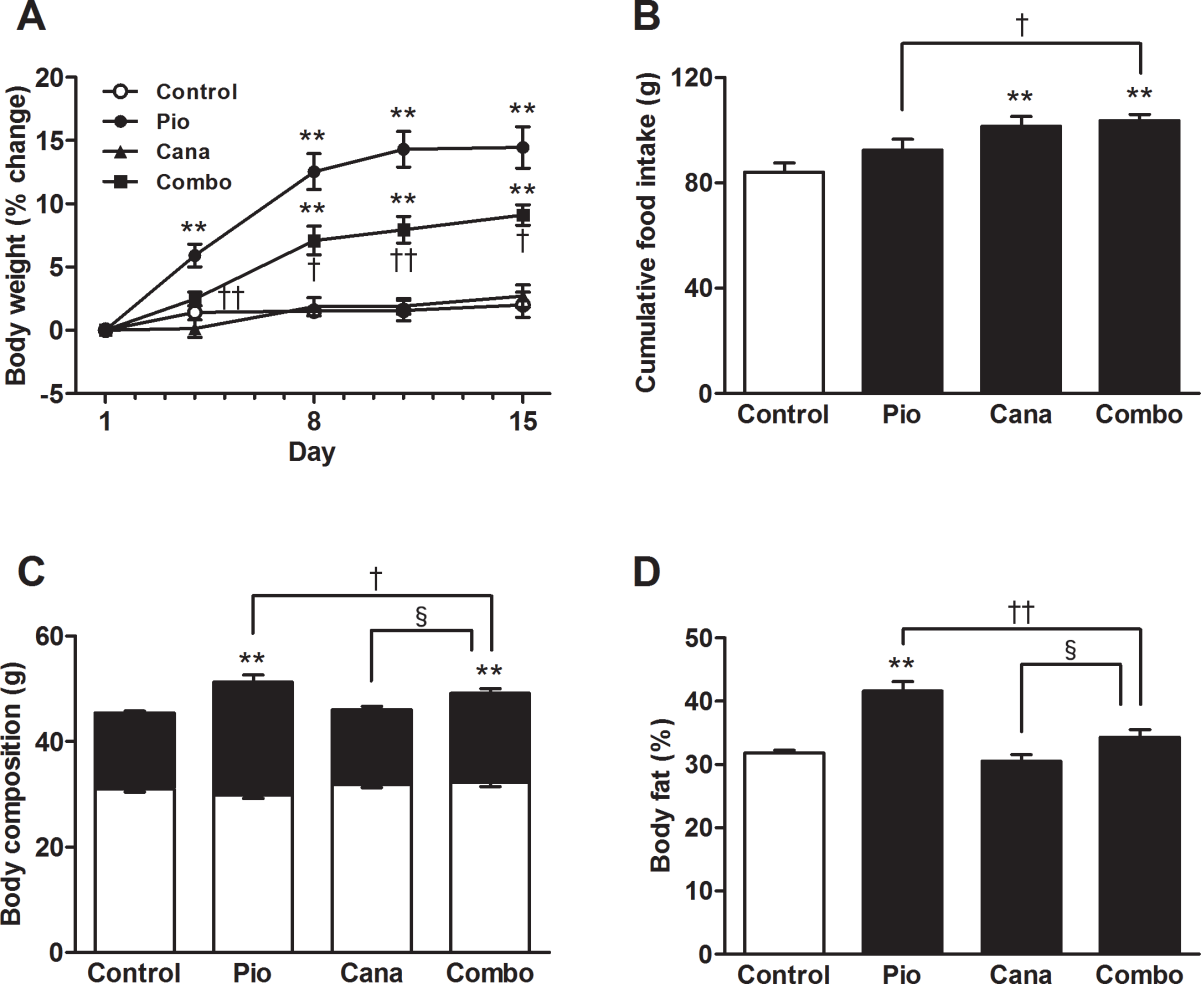
Body weight, food intake, and body composition by dual-energy X-ray absorptiometry (DEXA) in KK-A^y^ mice. A: Body weight percent change, B: Cumulative food intake, C: Body composition (lean mass, open column; fat mass, closed column), and D: Body fat. Canagliflozin (Cana; 0.01% w/w food admixture), pioglitazone (Pio; 0.01% w/w food admixture), and their combination (Combo) were administered for 2 weeks. Body composition and body fat were measured at the end of the study using the DEXA scanner. Data are expressed as mean ± SEM (n = 7). ** *P* < 0.01 vs. control, † *P* < 0.05, †† *P* < 0.01 vs. Pio, § *P* < 0.05 vs. Cana.

**Figure 3:**
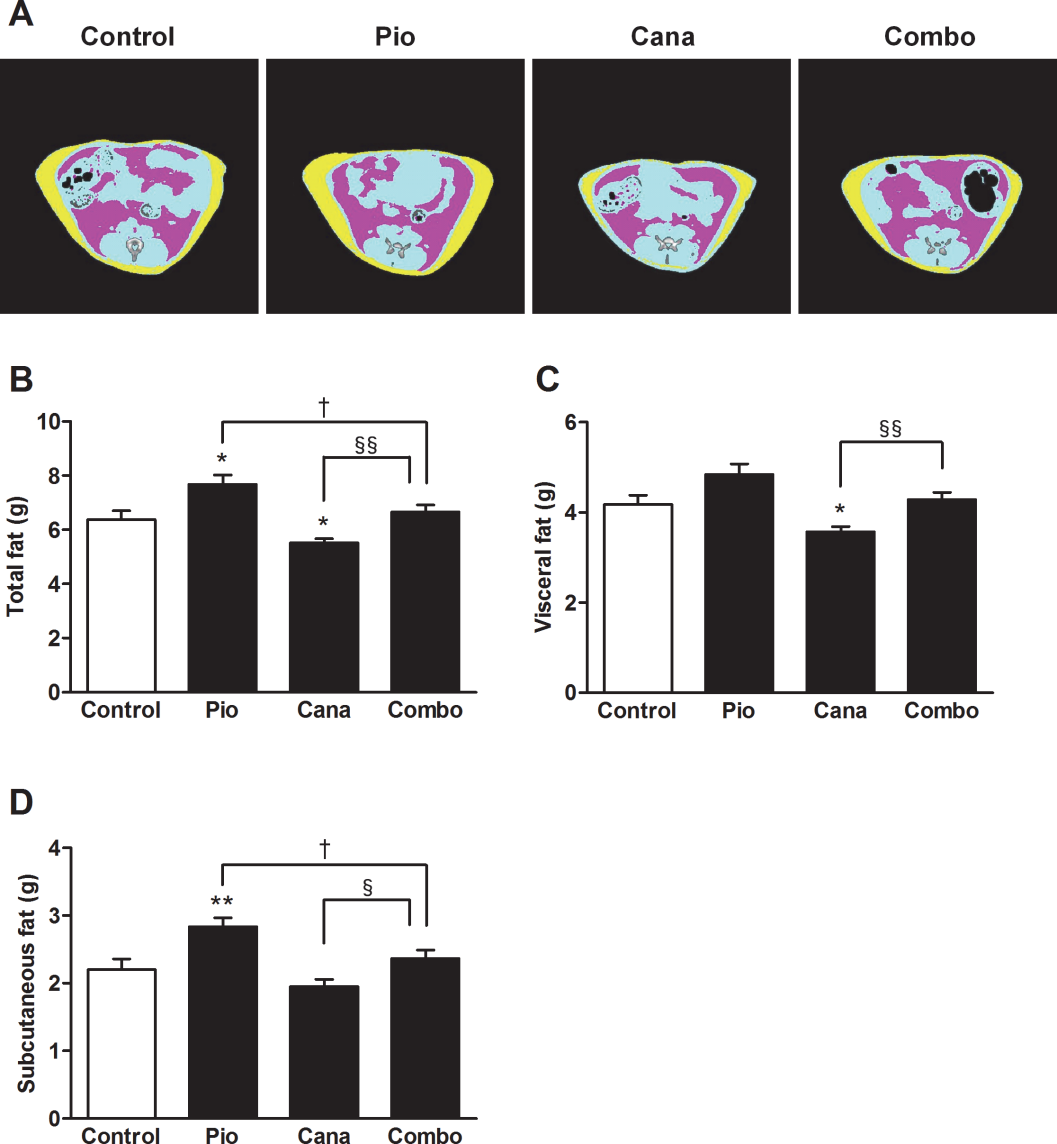
Body fat analysis by computed tomography (CT) in KK-A^y^ mice. A: Representative CT images (red, visceral fat; yellow, subcutaneous fat), B: Total fat, C: Visceral fat, and D: Subcutaneous fat. Canagliflozin (Cana; 0.01% w/w food admixture), pioglitazone (Pio; 0.01% w/w food admixture), and their combination (Combo) were administered for 2 weeks. Adipose tissue weight was measured at the end of the study using an experimental animal CT system. Data are expressed as mean ± SEM (n = 7–8). * *P* < 0.05, ** *P* < 0.01 vs. control, † *P* < 0.05 vs. Pio, § *P* < 0.05, §§ *P* < 0.01 vs. Cana.

**Figure 4:**
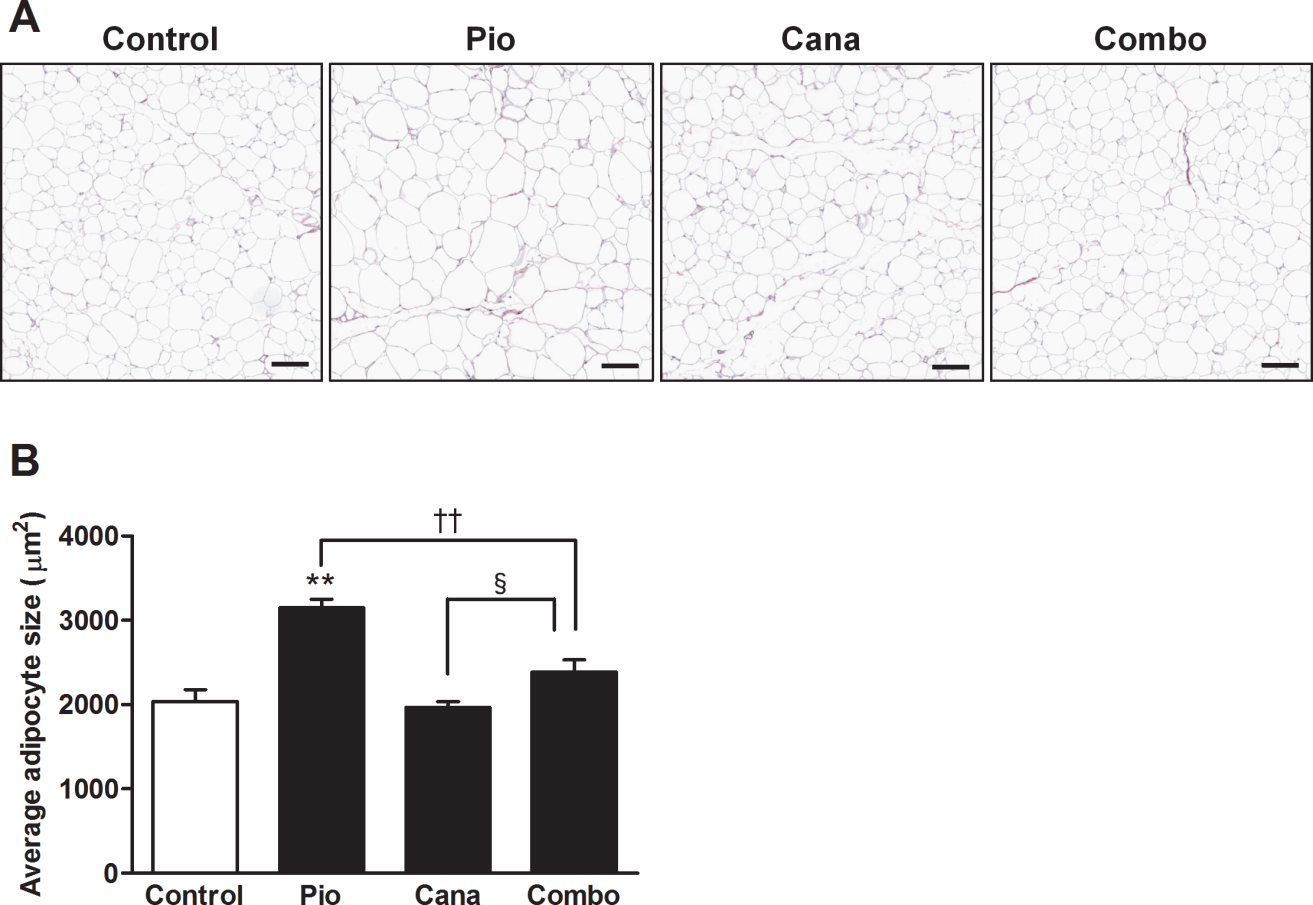
Histological analysis of adipose tissue in KK-A^y^ mice. A: Representative images of mesenteric adipose tissue and B: Average size of adipocytes. Canagliflozin (Cana; 0.01% w/w food admixture), pioglitazone (Pio; 0.01% w/w food admixture), and their combination (Combo) were administered for 2 weeks. Mesenteric adipose tissue was stained with elastic van Gieson without nuclear staining at the end of the study. The size of 500 adipocytes was analyzed using Image-Pro Plus software. Data are expressed as mean ± SEM (n = 8). ** *P* < 0.01 vs. control, †† *P* < 0.01 vs. Pio, § *P* < 0.05 vs. Cana. Scale bar = 100 μm.

The 2-week treatment with pioglitazone monotherapy induced significant body weight gain in the KK-A^y^ mice (Fig. 2A). Although canagliflozin monotherapy did not reduce body weight, the add-on treatment with canagliflozin significantly attenuated the pioglitazone-induced body weight gain. During the treatment period, canagliflozin monotherapy and the combination therapy each stimulated a slight increase in food intake (Fig. 2B). Body composition was measured at the end of the study by DEXA scanning. Canagliflozin monotherapy did not affect body composition; however, the add-on treatment markedly attenuated any increase in pioglitazone-induced fat mass without affecting lean mass (Fig. 2C and 2D). The effects on adiposity were further confirmed by CT scanning, which showed that pioglitazone-induced fat gain was markedly reduced by the add-on treatment (Fig. 3A-D). Furthermore, adipocyte size was analyzed in the mesenteric adipose tissue of the KK-A^y^ mice. Pioglitazone monotherapy clearly induced significant adipocyte hypertrophy (Fig. 4A and 4B). Canagliflozin monotherapy did not affect adipocyte size, whereas the add-on treatment significantly attenuated pioglitazone-induced adipocyte hypertrophy.

### Diabetes prevention in ZDF rats

Table 1 summarizes the effects of the combination of canagliflozin and pioglitazone on the development of diabetes in the prediabetic ZDF rats. At the beginning of the study, the ZDF rats exhibited marked hyperinsulinemia and subsequently developed progressive hyperglycemia as indicated by plasma glucose and HbA1c levels. Plasma insulin levels in the vehicle-treated ZDF rats gradually decreased to levels similar to those in the ZL rats. At the end of the study, pancreatic insulin content in the vehicle-treated ZDF rats was significantly lower than that in the ZL rats. Conversely, 6-week treatment with canagliflozin or pioglitazone monotherapy almost completely prevented the development of hyperglycemia; no further improvement was observed with the add-on treatment. The pancreatic insulin content after each monotherapy and the combination therapy was significantly higher than that of the vehicle-treated ZDF rats. Marked hyperinsulinemia was present in ZDF rats at the start of the treatment period. Pioglitazone monotherapy markedly reduced plasma insulin levels throughout the study period. In contrast, the insulin level was lower in the canagliflozin monotherapy group only at week 2. Add-on treatment of canagliflozin further reduced plasma insulin levels at weeks 4 and 6 compared with pioglitazone monotherapy.

**Table 1:** Effects of combination therapy on the development of diabetes in ZDF and ZL rats.

		ZDF Vehicle	ZDF Pio	ZDF Cana	ZDF Combo	ZL Vehicle
Plasma glucose (mg/dL)	0 wk	139.7 ± 2.3 ##	137.7 ± 2.4	140.5 ± 3.6	141.6 ± 3.1	125.6 ± 1.0
	2 wk	241.5 ± 20.8 ##	117.7 ± 3.3 **	143.8 ± 6.5 **	118.6 ± 3.8 ** §§	125.0 ± 5.3
	4 wk	412.6 ± 7.6 ##	128.1 ± 6.2 **	147.1 ± 5.5 **	132.2 ± 4.8 **	122.4 ± 1.4
	6 wk	518.4 ± 12.8 ##	143.9 ± 3.4 **	195.5 ± 11.8 **	142.6 ± 4.5 ** §§	127.5 ± 3.9
HbA1c (%)	0 wk	2.7 ± 0.03 #	2.7 ± 0.02	2.7 ± 0.02	2.7 ± 0.03	2.6 ± 0.02
	2 wk	3.6 ± 0.06 ##	2.8 ± 0.03 **	3.3 ± 0.04 **	3.0 ± 0.03 ** §§	3.0 ± 0.04
	4 wk	5.8 ± 0.20 ##	3.1 ± 0.03 **	3.5 ± 0.03 **	3.0 ± 0.04 ** §§	3.1 ± 0.03
	6 wk	8.1 ± 0.17 ##	3.5 ± 0.09 **	3.8 ± 0.08 **	3.4 ± 0.11 ** §§	3.4 ± 0.03
Plasma insulin (ng/mL)	0 wk	13.9 ± 1.3 ##	12.7 ± 1.1	13.8 ± 0.8	13.0 ± 0.9	0.4 ± 0.1
	2 wk	15.5 ± 2.0 ##	3.9 ± 0.3 **	7.7 ± 1.2 **	4.0 ± 0.4 ** §	1.0 ± 0.2
	4 wk	6.9 ± 1.3 ##	5.8 ± 0.6	9.4 ± 0.8	3.9 ± 0.5 † §§	0.9 ± 0.1
	6 wk	3.5 ± 0.2 ##	5.2 ± 0.4 **	12.5 ± 1.3 **	4.0 ± 0.3 † §§	1.7 ± 0.1
Pancreatic insulin (μg/g pancreas)	6 wk	10.4 ± 2.0 ##	99.7 ± 14.6 **	76.4 ± 11.5 **	79.3 ± 15.1 **	59.7 ± 7.9
Body weight (g)	0 wk	207.4 ± 2.4 ##	207.3 ± 2.1	207.8 ± 2.3	207.4 ± 2.0	162.0 ± 2.6
	2 wk	300.2 ± 2.9 ##	329.1 ±2.4 **	283.2 ± 2.2 **	305.1 ± 2.5 †† §§	223.4 ± 2.9
	4 wk	338.1 ± 7.2 ##	432.6 ± 3.8 **	349.8 ± 3.7	399.5 ± 4.3 ** †† §§	253.9 ± 4.1
	6 wk	363.3 ± 6.4 ##	533.6 ± 5.7 **	403.5 ± 4.8 **	480.7 ± 8.5 ** †† §§	281.4 ± 5.0
Food intake (g/day)	2 wk	27.7 ± 0.5 ##	36.0 ± 0.5 **	27.7 ± 0.2	34.0 ± 0.3 ** †† §§	17.4 ± 0.4
	4 wk	32.1 ± 0.8 ##	38.6 ± 0.6 **	33.5 ± 0.6	40.8 ± 0.9 ** §§	17.6 ± 0.4
	6 wk	37.8 ± 1.3 ##	38.3 ± 0.6	35.3 ± 0.6	38.5 ± 2.3	17.3 ± 0.4
Cumulative food intake (g)		1109.8 ± 22.6 ##	1298.2 ± 17.5 **	1126.4 ± 15.5	1337.7 ± 20.4 ** §§	613.8 ± 12.6

Canagliflozin (Cana; daily dose of 10 mg/kg), pioglitazone (Pio; daily dose of 10 mg/kg), and their combination (Combo) were administered by oral gavage for 6 weeks. All values were measured in the nonfasting state. Data are expressed as mean ± SEM (n = 8). # *P* < 0.05, ## *P* < 0.01 vs. ZL rats, ** *P* < 0.01 vs. vehicle-treated ZDF rats, † *P* < 0.05, †† *P* < 0.01 vs. Pio-treated ZDF rats, § *P* < 0.05, §§ *P* < 0.01 vs. Cana-treated ZDF rats.

### Body weight and adiposity in ZDF rats

**Figure 5:**
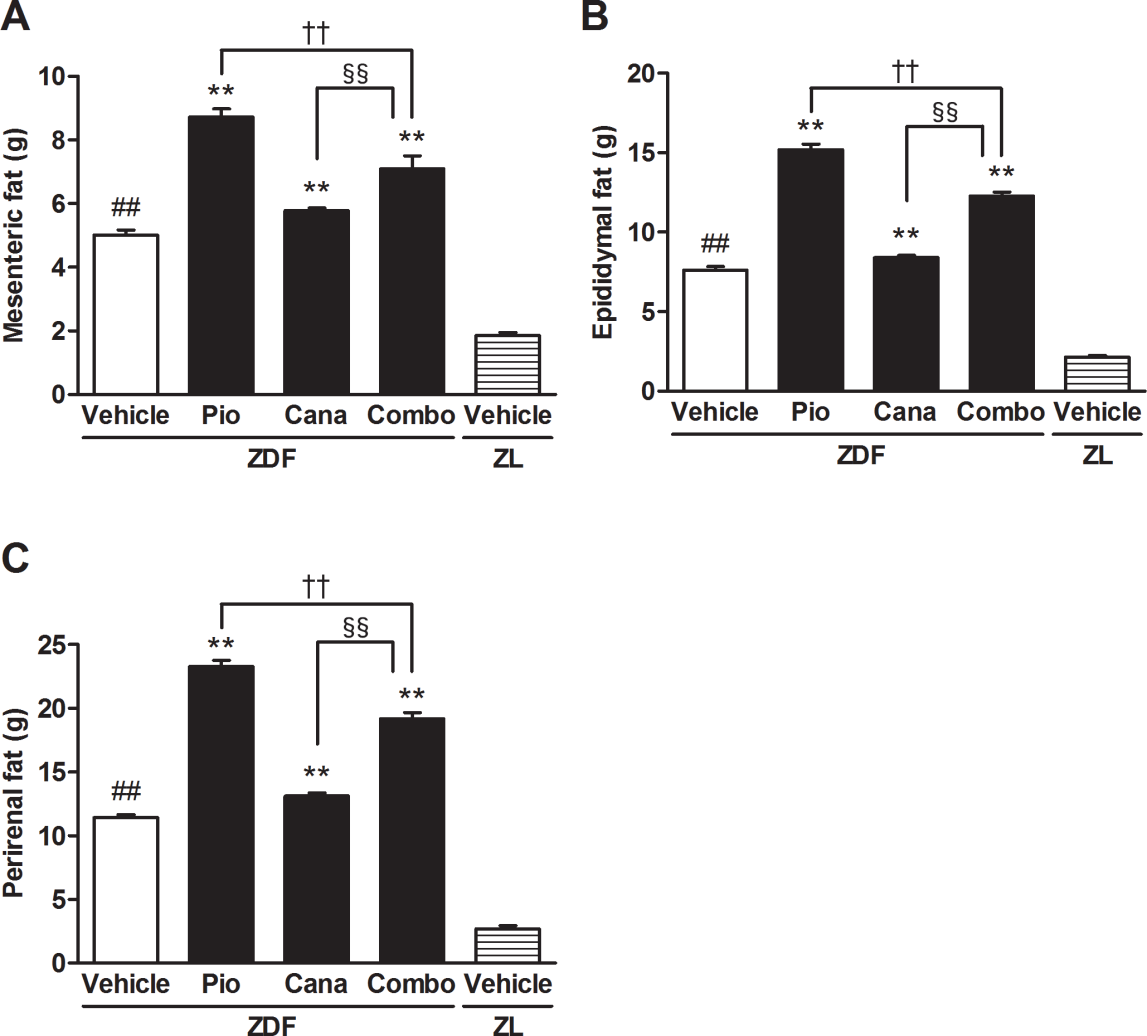
The adipose tissue weight of ZDF and ZL rats. A: Mesenteric fat, B: Epididymal fat, and C: Perirenal fat. Canagliflozin (Cana; daily dose of 10 mg/kg), pioglitazone (Pio; daily dose of 10 mg/kg), and their combination (Combo) were administered by oral gavage for 6 weeks. Adipose tissue weight was measured at the end of the study. Data are expressed as mean ± SEM (n = 8). ## *P* < 0.01 vs. ZL rats, ** *P* < 0.01 vs. vehicle-treated ZDF rats, †† *P* < 0.01 vs. Pio-treated ZDF rats, §§ *P* < 0.01 vs. Cana-treated ZDF rats.

Pioglitazone monotherapy significantly increased body weight throughout the study in the ZDF rats, whereas canagliflozin monotherapy slightly but significantly reduced body weight at week 2 and then increased it at week 6 (Table 1). However, the add-on treatment significantly attenuated pioglitazone-induced body weight gain. During the treatment period, pioglitazone monotherapy and the combination therapy significantly increased food intake, but canagliflozin monotherapy did not affect food intake (Table 1). At the end of the study, mesenteric, epididymal, and perirenal fat weights were measured. Pioglitazone monotherapy significantly increased all of these adipose tissue weights (Fig. 5A-C). Although canagliflozin monotherapy slightly increased these fat weights, the add-on treatment significantly attenuated the pioglitazone-induced adiposity.

### Insulin sensitivity in ZDF rats

**Figure 6:**
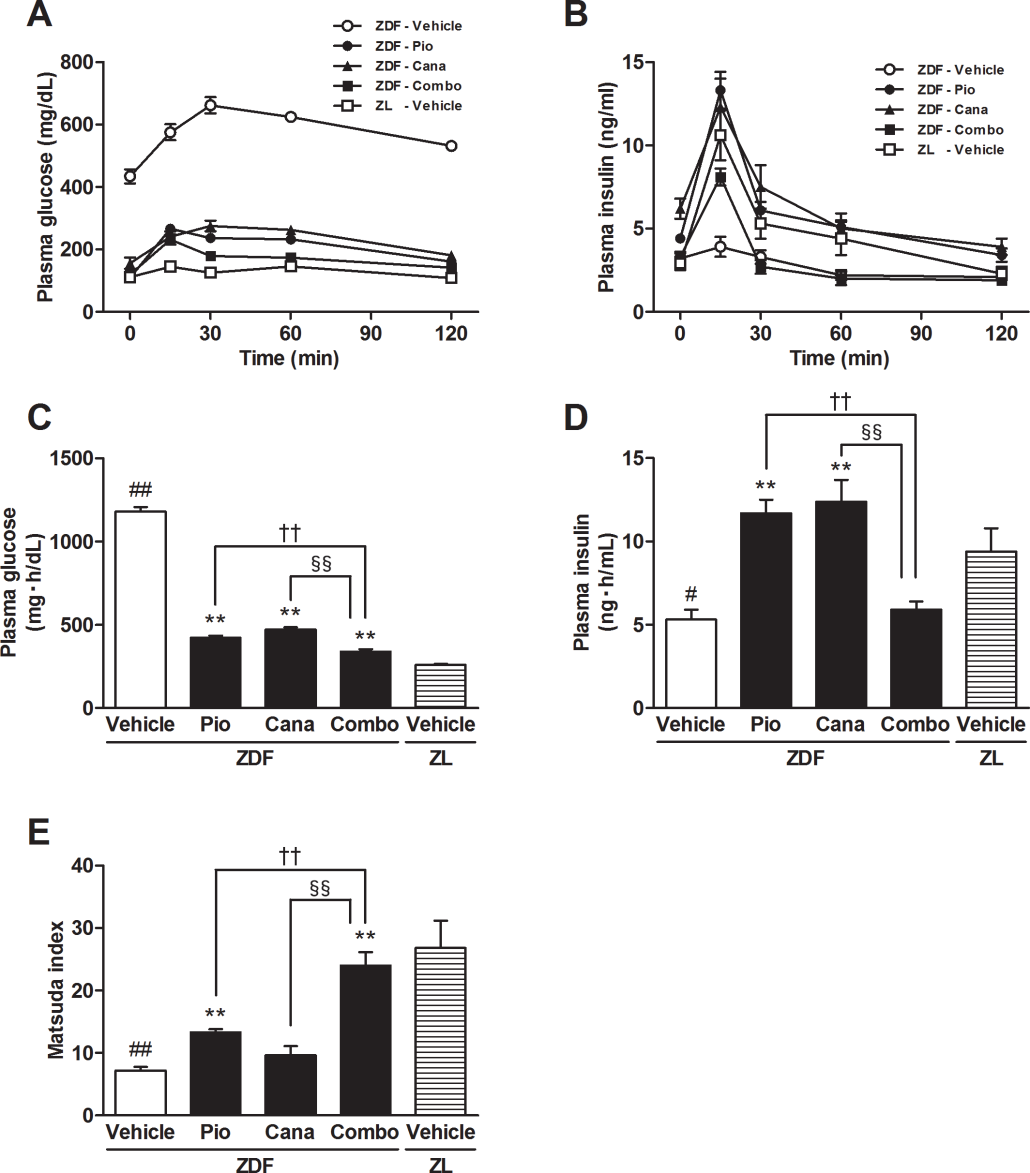
The oral glucose tolerance test (OGTT) and Matsuda index in ZDF and ZL rats. A: Plasma glucose, B: Plasma insulin, C: AUC_0–2 h_ of plasma glucose, D: AUC_0–2 h_ of plasma insulin, and E: Matsuda index. Canagliflozin (Cana; daily dose of 10 mg/kg), pioglitazone (Pio; daily dose of 10 mg/kg), and their combination (Combo) were administered by oral gavage for 6 weeks. OGTT was performed 2 days after the last dosing. Data are expressed as mean ± SEM (n = 8). # *P* < 0.05, ## *P* < 0.01 vs. ZL rats, * *P* < 0.05, ** *P* < 0.01 vs. vehicle-treated ZDF rats, †† *P* < 0.01 vs. Pio-treated ZDF rats, §§ *P* < 0.01 vs. Cana-treated ZDF rats.

OGTT was performed after the 6-week treatment in overnight fasted ZDF rats (Fig. 6A-D and Table 2). The AUC_0–2 h_ of plasma glucose levels during OGTT was significantly higher in the vehicle-treated ZDF rats than in the ZL rats. Each monotherapy significantly improved glucose intolerance, and further amelioration was achieved by the combination therapy. Although glucose-stimulated insulin secretion almost disappeared in the vehicle-treated ZDF rats, insulin secretion during the first 30 min was markedly improved by each monotherapy and the combination therapy. The AUC_0–2 h_ of plasma insulin levels was significantly higher with both monotherapies, but not with the combination therapy, reflecting the effects of basal insulin and insulin secretion during OGTT. The Matsuda index revealed that pioglitazone monotherapy significantly improved insulin sensitivity (Fig. 6E). Whereas canagliflozin monotherapy slightly but not significantly improved insulin sensitivity, the combination therapy significantly improved insulin sensitivity compared with the respective monotherapies.

**Table 2:** Effects of combination therapy on glucose and insulin excursions during OGTT in ZDF and ZL rats.

		**ZDF Vehicle**	**ZDF Pio**	**ZDF Cana**	**ZDF Combo**	**ZL Vehicle**
Plasma glucose (mg/dL)	0 min	434.3 ± 22.4 ##	109.8 ± 4.2 **	155.8 ± 18.2 **	119.4 ± 2.8 **	110.7 ± 3.7
	15 min	575.3 ± 25.9 ##	266.4 ± 8.8 **	241.1 ± 17.0 **	231.4 ± 16.0 **	144.7 ± 7.2
	30 min	661.8 ± 25.7 ##	236.1 ± 12.1 **	275.3 ± 17.1 **	178.8 ± 13.5 ** †† §§	125.8 ± 3.2
	60 min	624.2 ± 13.0 ##	232.0 ± 9.3 **	262.5 ± 11.9 **	173.4 ± 9.4 ** †† §§	145.6 ± 3.8
	120 min	531.7 ± 10.0 ##	160.9 ± 4.9 **	180.7 ± 10.9 **	141.7 ± 8.1 ** §	108.3 ± 2.4
Plasma insulin (ng/mL)	0 min	3.2 ± 0.3	4.4 ± 0.2 **	6.2 ± 0.6 **	3.3 ± 0.3 † §§	2.9 ± 0.4
	15 min	3.9 ± 0.6 ##	13.3 ± 1.1 **	12.3 ± 1.7 **	8.1 ± 0.5 ** †† §	10.6 ± 1.5
	30 min	3.3 ± 0.4	6.1 ± 0.5 **	7.5 ± 1.3 **	2.7 ± 0.4 †† §§	5.3 ± 0.9
	60 min	2.2 ± 0.3	5.1 ± 0.8 **	5.0 ± 0.5 **	2.0 ± 0.4 †† §§	4.4 ± 1.0
	120 min	2.1 ± 0.2	3.4 ± 0.4 *	3.9 ± 0.5 **	1.9 ± 0.2 †† §§	2.3 ± 0.4

Canagliflozin (Cana; daily dose of 10 mg/kg), pioglitazone (Pio; daily dose of 10 mg/kg), and their combination (Combo) were administered by oral gavage for 6 weeks. Overnight-fasted animals were subjected to OGTT 2 days after the last dosing. Data are expressed as mean ± SEM (n = 8). ## *P* < 0.01 vs. ZL rats, * *P* < 0.05, ** *P* < 0.01 vs. vehicle-treated ZDF rats, † *P* < 0.05, †† *P* < 0.01 vs. Pio-treated ZDF rats, § *P* < 0.05, §§ *P* < 0.01 vs. Cana-treated ZDF rats.

## Discussion

We demonstrated that the combination therapy with canagliflozin and pioglitazone markedly improved hyperinsulinemia in established diabetic KK-A^y^ mice, implying improved insulin sensitivity. Furthermore, the effects on insulin sensitivity were evaluated in the ZDF rats after early pharmacological intervention with the combination therapy. Several insulin resistance/sensitivity indices are calculated from plasma glucose and insulin in the fasting state and during OGTT. These include HOMA-IR and the Matsuda index, although the former may not represent appropriate insulin resistance in a subject with severely impaired β-cell function [22]. Conversely, the Matsuda index highly correlates with whole-body insulin sensitivity, as determined by the euglycemic insulin clamp technique, which is the gold standard for measuring insulin resistance [19]. In this context, we used the Matsuda index to estimate whole-body insulin sensitivity in the ZDF rats with progressive β-cell dysfunction [23]. We observed that the Matsuda index was significantly improved by early intervention with the combination therapy, which strongly suggests that combination therapy improves whole-body insulin sensitivity.

It is well known that the ability of insulin to stimulate glucose uptake and inhibit hepatic glucose production is impaired at an early stage in type 2 diabetes [24,25]. As a consequence, pancreatic β-cells respond by increasing insulin secretion to maintain normoglycemia [26]. In the presence of β-cell deficits, this compensatory response is progressively lost, yielding overt hyperglycemia [27]. Thereafter, sustained hyperglycemia leads to glucotoxicity, which further worsens insulin sensitivity and secretion [28–30]. Correction of hyperglycemia with a non-selective SGLT inhibitor, phlorizin, normalizes insulin sensitivity in partially pancreatectomized diabetic rats with moderate glucose intolerance [31] and non-insulin-dependent diabetic rats induced by neonatal streptozotocin [32]. In the present study, we observed that the combination therapy with a selective SGLT2 inhibitor, canagliflozin, and an insulin sensitizer, pioglitazone, further improved glycemic control in the KK-A^y^ mice with established diabetes. This finding implies that insulin resistance may be improved by the prevention of glucotoxicity. On the other hand, early intervention with either canagliflozin or pioglitazone monotherapy almost completely prevented the progressive development of hyperglycemia in the ZDF rats. At the end of the study, each monotherapy and the combination therapy maintained pancreatic insulin content and glucose-stimulated insulin secretion during OGTT at levels comparable with those in the ZL rats, probably due to the attenuation of glucotoxicity. These results strongly suggest that early intervention is favorable to maintain pancreatic β-cell function. This is consistent with a previous report that states early intervention with antidiabetic agents can prevent type 2 diabetes [33]. In addition, whereas no further improvement in glycemic control was achieved by the add-on treatment with canagliflozin, the combination therapy significantly improved whole-body insulin sensitivity compared with the respective monotherapies. Taken together, these results imply the existence of mechanisms independent of blood glucose lowering that underlie the improvement of insulin sensitivity observed with the combination therapy.

As obesity is a risk factor for developing insulin resistance [4,5], control of body weight is important for the prevention and treatment of type 2 diabetes. In the present study, we observed that add-on treatment with canagliflozin significantly attenuated pioglitazone-induced body weight and fat gain. Such a response may partly contribute to the improvement of insulin sensitivity independent of blood glucose lowering. This attenuation of weight gain was explained by enhanced urinary calorie loss via canagliflozin-induced UGE, as observed in KK-A^y^ mice. In addition, our previous study showed that canagliflozin monotherapy significantly reduces the respiratory exchange ratio in DIO mice [15], suggesting that increased fatty acid metabolism may account for the reduction in body weight gain.

Certain limitations of this study should be considered when interpreting the results. First, body weight reduction may be attenuated by compensatory hyperphagia in response to increased UGE. This has been previously reported in DIO rats treated with the SGLT2 inhibitor dapagliflozin [34]. We observed that canagliflozin induced compensatory hyperphagia in the KK-A^y^ mice, possibly explaining the lack of reduction of body weight gain with canagliflozin monotherapy. Second, plasma insulin may affect body weight via its anabolic effects. We recently reported that plasma insulin levels are transiently elevated at 6–7 weeks of age in vehicle-treated ZDF rats, and then gradually decrease because of pancreatic exhaustion [35]. In addition, the above study showed that canagliflozin attenuates the pancreatic exhaustion and subsequently increases plasma insulin levels, resulting in body weight gain. In the present study, canagliflozin monotherapy induced this paradoxical weight gain without compensatory hyperphagia. In contrast, the pioglitazone monotherapy induced body weight gain despite lower plasma insulin levels via its insulin-sensitizing effects. Thus, canagliflozin and pioglitazone both increase body weight gain in ZDF rats, although plasma insulin levels differ between canagliflozin- and pioglitazone-treated animals because of their different modes of action. The combination of these two drugs achieved the lowest plasma insulin levels among the drug treatment groups and attenuated pioglitazone-induced body weight gain. Finally, there are differences between rodent and human type 2 diabetes. Compensatory hyperphagia was observed in canagliflozin-treated KK-A^y^ mice but was not reported in clinical trials of canagliflozin. Furthermore, the SGLT2 inhibitor-induced increase in plasma insulin levels and subsequent weight gain is limited to animal models such as ZDF rats and db/db mice, which develop severe pancreatic dysfunction within a short period [35–37].

In conclusion, our findings suggest that combination therapy with canagliflozin and pioglitazone markedly improves hyperglycemia and hyperinsulinemia in obesity-associated type 2 diabetes. Furthermore, the addition of canagliflozin to pioglitazone therapy improves insulin sensitivity partly by preventing glucotoxicity and, at least partly, by attenuating pioglitazone-induced body weight gain. This combination therapy may represent a valuable option for the treatment and prevention of obesity-associated type 2 diabetes.

## References

[1] Ogden CL, Carroll MD, Curtin LR, McDowell MA, Tabak CJ, et al. (2006) Prevalence of overweight and obesity in the United States, 1999–2004. Jama 295: 1549–1555. 16595758 10.1001/jama.295.13.1549

[2] Swinburn BA, Sacks G, Hall KD, McPherson K, Finegood DT, et al. (2011) The global obesity pandemic: shaped by global drivers and local environments. Lancet 378: 804–814. 10.1016/S0140-6736(11)60813-1 21872749

[3] Withrow D, Alter DA (2011) The economic burden of obesity worldwide: a systematic review of the direct costs of obesity. Obes Rev 12: 131–141. 20122135 10.1111/j.1467-789X.2009.00712.x

[4] Everson SA, Goldberg DE, Helmrich SP, Lakka TA, Lynch JW, et al. (1998) Weight gain and the risk of developing insulin resistance syndrome. Diabetes Care 21: 1637–1643. 9773723 10.2337/diacare.21.10.1637

[5] Kahn BB, Flier JS (2000) Obesity and insulin resistance. J Clin Invest 106: 473–481. 10953022 10.1172/JCI10842 PMC380258

[6] Kahn SE, Hull RL, Utzschneider KM (2006) Mechanisms linking obesity to insulin resistance and type 2 diabetes. Nature 444: 840–846. 17167471 10.1038/nature05482

[7] Wild S, Roglic G, Green A, Sicree R, King H (2004) Global prevalence of diabetes: estimates for the year 2000 and projections for 2030. Diabetes Care 27: 1047–1053. 15111519 10.2337/diacare.27.5.1047

[8] Nyenwe EA, Jerkins TW, Umpierrez GE, Kitabchi AE (2011) Management of type 2 diabetes: evolving strategies for the treatment of patients with type 2 diabetes. Metabolism 60: 1–23. 21134520 10.1016/j.metabol.2010.09.010 PMC3746516

[9] Ali MK, Bullard KM, Saaddine JB, Cowie CC, Imperatore G, et al. (2013) Achievement of goals in U.S. diabetes care, 1999–2010. N Engl J Med 368: 1613–1624. 23614587 10.1056/NEJMsa1213829

[10] Bergenstal RM, Bailey CJ, Kendall DM (2010) Type 2 diabetes: assessing the relative risks and benefits of glucose-lowering medications. Am J Med 123: 374 e 379–318 10.1016/j.amjmed.2009.07.017 20362759

[11] Nesto RW, Bell D, Bonow RO, Fonseca V, Grundy SM, et al. (2003) Thiazolidinedione use, fluid retention, and congestive heart failure: a consensus statement from the American Heart Association and American Diabetes Association. October 7, 2003. Circulation 108: 2941–2948. 14662691 10.1161/01.CIR.0000103683.99399.7E

[12] Devineni D, Morrow L, Hompesch M, Skee D, Vandebosch A, et al. (2012) Canagliflozin improves glycaemic control over 28 days in subjects with type 2 diabetes not optimally controlled on insulin. Diabetes Obes Metab 14: 539–545. 10.1111/j.1463-1326.2012.01558.x 22226086

[13] Rosenstock J, Aggarwal N, Polidori D, Zhao Y, Arbit D, et al. (2012) Dose-ranging effects of canagliflozin, a sodium-glucose cotransporter 2 inhibitor, as add-on to metformin in subjects with type 2 diabetes. Diabetes Care 35: 1232–1238. 10.2337/dc11-1926 22492586 PMC3357223

[14] Arakawa K, Ishihara T, Oku A, Nawano M, Ueta K, et al. (2001) Improved diabetic syndrome in C57BL/KsJ-db/db mice by oral administration of the Na(+)-glucose cotransporter inhibitor T-1095. Br J Pharmacol 132: 578–586. 11159708 10.1038/sj.bjp.0703829 PMC1572576

[15] Liang Y, Arakawa K, Ueta K, Matsushita Y, Kuriyama C, et al. (2012) Effect of canagliflozin on renal threshold for glucose, glycemia, and body weight in normal and diabetic animal models. PLoS One 7: e30555 10.1371/journal.pone.0030555 22355316 PMC3280264

[16] Sha S, Devineni D, Ghosh A, Polidori D, Chien S, et al. (2011) Canagliflozin, a novel inhibitor of sodium glucose co-transporter 2, dose dependently reduces calculated renal threshold for glucose excretion and increases urinary glucose excretion in healthy subjects. Diabetes Obes Metab 13: 669–672. 10.1111/j.1463-1326.2011.01406.x 21457428

[17] Inzucchi SE, Bergenstal RM, Buse JB, Diamant M, Ferrannini E, et al. (2012) Management of hyperglycemia in type 2 diabetes: a patient-centered approach: position statement of the American Diabetes Association (ADA) and the European Association for the Study of Diabetes (EASD). Diabetes Care 35: 1364–1379. 22517736 10.2337/dc12-0413 PMC3357214

[18] Forst T, Guthrie R, Goldenberg R, Yee J, Vijapurkar U, et al. (2014) Efficacy and safety of canagliflozin over 52 weeks in patients with type 2 diabetes on background metformin and pioglitazone. Diabetes Obes Metab 16: 467–477. 10.1111/dom.12273 24528605 PMC4237547

[19] Matsuda M, DeFronzo RA (1999) Insulin sensitivity indices obtained from oral glucose tolerance testing: comparison with the euglycemic insulin clamp. Diabetes Care 22: 1462–1470. 10480510 10.2337/diacare.22.9.1462

[20] Finkelstein A, Kunis G, Seksenyan A, Ronen A, Berkutzki T, et al. (2011) Abnormal changes in NKT cells, the IGF-1 axis, and liver pathology in an animal model of ALS. PLoS One 6: e22374 10.1371/journal.pone.0022374 21829620 PMC3149057

[21] Jia L, Xing J, Ding Y, Shen Y, Shi X, et al. (2013) Hyperuricemia causes pancreatic beta-cell death and dysfunction through NF-kappaB signaling pathway. PLoS One 8: e78284 10.1371/journal.pone.0078284 24205181 PMC3808354

[22] Muniyappa R, Lee S, Chen H, Quon MJ (2008) Current approaches for assessing insulin sensitivity and resistance in vivo: advantages, limitations, and appropriate usage. Am J Physiol Endocrinol Metab 294: E15–26. 17957034 10.1152/ajpendo.00645.2007

[23] Etgen GJ, Oldham BA (2000) Profiling of Zucker diabetic fatty rats in their progression to the overt diabetic state. Metabolism 49: 684–688. 10831184 10.1016/s0026-0495(00)80049-9

[24] Mitrakou A, Kelley D, Mokan M, Veneman T, Pangburn T, et al. (1992) Role of reduced suppression of glucose production and diminished early insulin release in impaired glucose tolerance. N Engl J Med 326: 22–29. 1727062 10.1056/NEJM199201023260104

[25] Weyer C, Bogardus C, Mott DM, Pratley RE (1999) The natural history of insulin secretory dysfunction and insulin resistance in the pathogenesis of type 2 diabetes mellitus. J Clin Invest 104: 787–794. 10491414 10.1172/JCI7231 PMC408438

[26] Prentki M, Nolan CJ (2006) Islet beta cell failure in type 2 diabetes. J Clin Invest 116: 1802–1812. 16823478 10.1172/JCI29103 PMC1483155

[27] Butler AE, Janson J, Bonner-Weir S, Ritzel R, Rizza RA, et al. (2003) Beta-cell deficit and increased beta-cell apoptosis in humans with type 2 diabetes. Diabetes 52: 102–110. 12502499 10.2337/diabetes.52.1.102

[28] Del Prato S (2009) Role of glucotoxicity and lipotoxicity in the pathophysiology of Type 2 diabetes mellitus and emerging treatment strategies. Diabet Med 26: 1185–1192. 10.1111/j.1464-5491.2009.02847.x 20002468

[29] Nawano M, Oku A, Ueta K, Umebayashi I, Ishirahara T, et al. (2000) Hyperglycemia contributes insulin resistance in hepatic and adipose tissue but not skeletal muscle of ZDF rats. Am J Physiol Endocrinol Metab 278: E535–543. 10710509 10.1152/ajpendo.2000.278.3.E535

[30] Fujimoto Y, Torres TP, Donahue EP, Shiota M (2006) Glucose toxicity is responsible for the development of impaired regulation of endogenous glucose production and hepatic glucokinase in Zucker diabetic fatty rats. Diabetes 55: 2479–2490. 16936196 10.2337/db05-1511

[31] Rossetti L, Smith D, Shulman GI, Papachristou D, DeFronzo RA (1987) Correction of hyperglycemia with phlorizin normalizes tissue sensitivity to insulin in diabetic rats. J Clin Invest 79: 1510–1515. 3571496 10.1172/JCI112981 PMC424427

[32] Blondel O, Bailbe D, Portha B (1990) Insulin resistance in rats with non-insulin-dependent diabetes induced by neonatal (5 days) streptozotocin: evidence for reversal following phlorizin treatment. Metabolism 39: 787–793. 2198430 10.1016/0026-0495(90)90120-2

[33] DeFronzo RA, Abdul-Ghani M (2011) Type 2 diabetes can be prevented with early pharmacological intervention. Diabetes Care 34 Suppl 2: S202–209. 21525456 10.2337/dc11-s221 PMC3632162

[34] Devenny JJ, Godonis HE, Harvey SJ, Rooney S, Cullen MJ, et al. (2012) Weight loss induced by chronic dapagliflozin treatment is attenuated by compensatory hyperphagia in diet-induced obese (DIO) rats. Obesity (Silver Spring) 20: 1645–1652. 10.1038/oby.2012.59 22402735

[35] Kuriyama C, Xu JZ, Lee SP, Qi J, Kimata H, et al. (2014) Analysis of the Effect of Canagliflozin on Renal Glucose Reabsorption and Progression of Hyperglycemia in Zucker Diabetic Fatty Rats. J Pharmacol Exp Ther. 10.1124/jpet.114.217992 25216746

[36] Fujimori Y, Katsuno K, Nakashima I, Ishikawa-Takemura Y, Fujikura H, et al. (2008) Remogliflozin etabonate, in a novel category of selective low-affinity sodium glucose cotransporter (SGLT2) inhibitors, exhibits antidiabetic efficacy in rodent models. J Pharmacol Exp Ther 327: 268–276. 10.1124/jpet.108.140210 18583547

[37] Kurosaki E, Ogasawara H (2013) Ipragliflozin and other sodium-glucose cotransporter-2 (SGLT2) inhibitors in the treatment of type 2 diabetes: preclinical and clinical data. Pharmacol Ther 139: 51–59. 10.1016/j.pharmthera.2013.04.003 23563279

